# Targeting p53 by small molecule p53 activators in multiple myeloma

**DOI:** 10.1186/1756-8722-5-S1-A7

**Published:** 2012-04-25

**Authors:** Manujendra N Saha, Yijun Yang, Hong Chang

**Affiliations:** 1 Dept. of Laboratory Hematology, University Health Network, Dept. of Laboratory Medicine and Pathobiology, University of Toronto, Ontario, Canada

## 

For the past three decades of research, p53 has been identified as one of the most targetable molecules for developing anticancer treatments. This tumor suppressor protein is involved in apoptosis, cell cycle arrest and senescence. Impairment of p53 function in tumors occurs either as a result of mutations in the *TP53* gene itself or the abrogation of signaling pathways regulating p53 that are required to exert its cellular function [1]. MDM2 is a transcriptional target of p53, which creates an important negative feedback loop that controls the activity of p53 in response to stress. The MDM2 E3 ubiquitin ligase tightly regulates p53 by targeting it for ubiquitin-dependent proteasomal degradation [2]. In experimental models, disrupting the MDM2–p53 interaction restored p53 function and sensitized tumors to chemotherapy or radiotherapy. This strategy could be particularly beneficial in treating cancers that rarely harbor *TP53* mutations/deletions; for example, hematologic malignancies such as multiple myeloma (MM), chronic lymphocytic leukemia (CLL), acute lymphoblastic leukemia (ALL), and acute myeloid leukemia (AML) [1,3].

The most well studied small-molecule inhibitor of the MDM2–p53 complex is nutlin. Nutlins are a group of cis-imidazoline analogs that have a high binding potency and selectivity for MDM2 and are being tested in phase I clinical trials in patients with hematologic neoplasms and advanced solid tumors [4]. p53-mediated effects of nutlin, such as induction of cell cycle arrest and/or apoptosis, have been demonstrated in cancers characterized by non-mutated *TP53*, including B-CLL, AML, and ALL [1]. Moreover, it can inhibit tumor growth in a non-genotoxic manner in xenografted tumor mice [1]. We have provided the evidence that nutlin mediated apoptosis can be mediated by both extrinsic and intrinsic pathways. Importantly, our study demonstrated that nutlin can utilize both p53-transcription-depepdent and transcription-independent mechanisms to trigger p53-mediated apoptosis suggesting that transcriptional and mitochondrial functions of p53 are equally important for nutlin-triggered apoptosis in MM cells [5].

Another activator of the p53 pathway is the small molecule RITA (reactivation of p53 and induction of tumor cell apoptosis) which is not yet in clinical trials. RITA acts differently to the nutlins by binding directly to its proposed p53 (rather than MDM2) binding site and blocking its ability to interact with MDM2 [6]. However, there appears other mechanisms by which RITA increases p53 activity in cells, since there is evidence that RITA can bind to multiple proteins and activate DNA damage response pathways [7]. We previously evaluated anti-myeloma activity of RITA and showed that RITA-induced apoptosis in MM cells is mediated by caspase-dependent extrinsic pathways. On the other hand, nonmalignant cells seem to tolerate RITA and no significant toxic effects have been observed in normal cells [8]. More recently, our study reveals that RITA induced p53-dependent apoptosis of MM cells is mediated by targeting JNK or its upstream targets. Both genetic and pharmacological Inhibition of JNK activation resulted in inhibition of activation of p53 and induction of apoptosis by RITA [9]. Moreover, RITA shows preclinical activity for retardation of tumor growth and prolongation of survival in MM mouse xenograft models. In addition, we also demonstrated potential synergistic cytotoxic responses of RITA in combination with nutlin [8] or with the JNK activators dexamethasone or 2-Cyano-3,12-dioxooleana-1,9-dien-28 oic Acid (CDDO) [9]; or of nutlin in combination with velcade, a proteasome inhibitor [10]. Our results indicated a novel mechanism for RITA in JNK signaling and p53-mediated apoptosis in MM cells and provided a preclinical framework for evaluating RITA in clinical trials for the treatment of MM.

Our studies underscore the tremendous potential of these two small molecules for enhancing our understanding of the intricate complexities between different networks of cell death as well as for therapeutic induction of apoptosis in myeloma cells. Further, nutlin or RITA in combination with other available therapeutic agents and also as single agents present a promising novel approach for p53-targeted therapies of MM, which warrants further exploitation.
